# Assessment of Pulp Vitality in Multirooted Teeth With Advanced Periodontal Disease: A Clinical and Histological Study

**DOI:** 10.7759/cureus.33298

**Published:** 2023-01-03

**Authors:** Srivainavi Arulmari, Ashwini Athul, Mahalingam Bhuvaneswari, Anitha Vijayarangan, Agila Elumalai, Shanmugam Muthukali, Ashwath Balachandran

**Affiliations:** 1 Periodontics, Chettinad Dental College and Research Institute, Chennai, IND; 2 Oral and Maxillofacial Pathology, Chettinad Dental College and Research Institute, Chennai, IND

**Keywords:** stage iv periodontitis, stage iii periodontitis, chronic periodontitis, histopathology (hp), apical periodontitis, gingival recession, perio, pulp, endo perio lesions

## Abstract

Background/purpose: The relationship between endodontic and periodontal lesions remains a controversy. Their diagnosis is often difficult and requires an interdisciplinary approach to rule out the cause and provide appropriate treatment. Periodontitis as an etiology of pulpal necrosis and irreversible pulpitis has been a hypothetical concept. Thus, the aim of this study was to assess the non-carious teeth extracted due to periodontitis both clinically and histologically to understand the possible association between periodontitis and its effect on pulp vitality.

Materials and methods: The study consisted of 60 teeth, of which 20 were extracted due to orthodontic requirements (control group) and 40 were extracted due to periodontitis (test group), which was further subclassified based on the presence or absence of gingival recession. Clinically, the teeth were categorized as non-vital after testing them with the electronic pulp tester (EPT). Later, these teeth were sectioned, and histopathological analysis was done to detect the presence of lateral or accessory canals.

Results: The results showed that there were mild to moderate deteriorative changes in the pulp in the periodontitis group without a gingival recession and moderate to severe changes in the pulp in the periodontitis group with a gingival recession.

Conclusion: There exists a possible deteriorative effect on pulp vitality as a consequence of periodontitis, even when the vitality of the pulp remains unaffected by dental caries.

## Introduction

Periodontal disease along with dental caries constitute the two most common dental conditions worldwide, affecting patients of all ages and genders. The varied symptoms of periodontitis include, among others, gingival bleeding, suppuration, pockets, attachment loss, mobility, furcation involvement, and gingival recession. Advanced caries involves the pulp, necessitating root canal treatment, which is easily identified and performed. However, pulpal involvement can also occur without the presence of dental caries due to advanced periodontal disease. If only periodontal therapy is performed without identification of pulpal involvement and subsequent pulpal therapy, it can lead to failure of the treatment.

Simring and Goldberg discovered a link between the pulp and the periodontium in the early embryonic stage of development in 1964 [1]. The derivatives of ectomesenchymal cells, such as the dental papilla and the dental sac, act as the precursors for the pulp and the periodontium, respectively. During the late bell stage of tooth development, Hertwig’s epithelial root sheath (HERS) separates the dental papilla and the dental sac, except at the future apical foramen. Later, when the HERS disintegrates, communication is established between the pulp and the periodontium via the apical foramen. The vascular channels in the apical foramen and aberrant accessory canals are responsible for this primitive relationship between the two tissues [2].

The pulp and the periodontium exhibit an embryonic and functional interrelationship that is not only helpful in maintaining physiological integrity but also enables the pathway for pathological invasions. This occurs due to the apical extension of pulpal infection into the periapical region, resulting in a periodontal pocket as a sequel of periodontal destruction or the extension of periodontal infection to the root canal chamber and pulp via the lateral canals, accessory canals, and apical foramen [1,2].

Didilescu et al. documented a possible association between six bacteria in the Endodontic-periodontal lesions using Polymerase Chain Reaction (PCR) and DNA-DNA hybridization [3]. These include Parvimonas micra, Fusobacterium nucleatum, Eikkenella corrodens, Campylobacter rectum, Eubacterium nodatum, and Capnocytophaga sputigena. Although the common etiology is a bacterial infection, iatrogenic root perforations, trauma, and vertical root fracture could also debride the root of its protective cementum layer, resulting in endodontic and periodontal lesions [2].

A state of anatomical relationship is maintained between the pulp and the periodontium throughout life, and thus, it is legible to say that if one structure is affected, it might affect the other, or the structures can be affected due to independent etiopathogenesis. Langeland et al. stated that the teeth affected by chronic periodontitis exhibited pathologic changes in the pulp such as inflammatory alterations, localized necrosis, calcifications, root resorption, and the deposition of reactionary dentin due to the spread of noxious inflammatory substances in a retrograde manner through the lateral and accessory canals [4]. Similarly, Wan et al. reported that the severity of periodontitis had a considerable effect on pulpal health [5]. Ghoddosi et al. stated that increased periodontal pocket depth as a consequence of severe periodontitis resulted in atrophied blood vessels in the pulp and increased pulpal calcifications [6].

There may be varied opinions among researchers, owing to differences in periodontal diagnostic criteria, difficulties in pulpal tissue fixation, a lack of healthy controls, or a lack of clear histological criteria for endodontic-periodontal lesions. With this literature background, we framed a study to assess the clinical and histologic findings of pulp vitality in multirooted teeth in patients with advanced periodontal diseases and also aim to correlate the findings with the presence of lateral or accessory canals.

## Materials and methods

The present research was designed as a case-control study. The study was approved by the Institutional Human Ethics Committee, Chettinad Academy of Research and Education, Kelambakkam (IHEC-I/0327/21). All the participants enrolled in the study were informed about the research protocol with informed consent registered before the initiation of the study. This study was conducted between January 2022 and April 2022.

Sample size calculation

Using the sample sizes of similar studies, the goodness-of-fit test was done, and the minimum sample size required to perform this study was calculated as 48, for which the power of the study was 0.95. Hence, we decided to incorporate 60 samples to enhance the validity of the study, which is 12 more than the minimum sample size required.

Also, to avoid any disparity in the distribution of the maxillary and mandibular molars, a chi-square analysis was done. The chi-square statistic was calculated as 0.4762. The p-value was 0.490153 (not significant at p < 0.05). Thus, the distribution of maxillary and mandibular molars in subgroups A and B did not vary significantly, nor did it impact the results of the study.

Subject population and selection

The selection of subjects with advanced periodontitis for the study group, clinical parameter recordings, and extractions were carried out in the Department of Periodontology, Chettinad Dental College and Research Institute. The histological assessments and evaluations were carried out in the Department of Oral and Maxillofacial Pathology and Oral Microbiology, Chettinad Dental College, and Research Institute. The subjects in the control group were selected from patients requiring extractions for orthodontic indications who reported to the Department of Orthodontics, Chettinad Dental College and Research Institute. The inclusion criteria were systemically healthy individuals of age 18-55 years, patients requiring extraction for orthodontic reasons (control group), patients with advanced periodontitis (Stage III or IV, Grade B or Grade C) with multirooted teeth of hopeless prognosis that includes [7], non-carious teeth with periodontitis, teeth with grade III mobility, presence of grade III or IV furcation involvement, Miller class III or IV gingival recession, radiographic presence of bone loss extending to or beyond the apical third of the root. Patients presenting with systemic disorders such as uncontrolled diabetes, cardiac illness, liver diseases, bleeding disorders and immunosuppression, current use of tobacco and alcohol, and pregnant and lactating women were excluded from the study.

Study design

This research was planned as a case-control study. All the subjects were divided into three groups based on the inclusion and exclusion criteria. Based on the 2018 Classification of Periodontal Diseases and Conditions (American Academy of Periodontology), the study population was assigned to stage III and stage IV periodontitis. The study groups were further subclassified into group A and group B based on the presence or absence of gingival recession.

Control group

The control group included 20 non-carious, periodontally healthy maxillary and mandibular premolars that needed extraction for orthodontic purposes.

Study group

The teeth selected in the study group involved 12 maxillary molars and 28 mandibular molars, with one or more of the following clinical findings such as probing pocket depth (PPD) of more than 7 mm, clinical attachment loss (CAL) of more than 5 mm, grade III/IV furcation involvement, Miller class III/IV gingival recession, and grade II/III mobility. These teeth were assigned a hopeless prognosis and were indicated for extraction.

Sub-Group A

Non-carious molars, which included five maxillary molars and 15 mandibular molars, that are indicated for extraction due to one or more of the following clinical findings, such as probing pocket depth >7 mm, clinical attachment loss of >5 mm, grade III/IV furcation involvement, grade II/III mobility with no evidence of gingival recession, are selected for sub-group A.

Sub-Group B

Non-carious molars, which included 7 maxillary molars and 13 mandibular molars, that are indicated for extraction due to one or more of the following clinical findings, such as probing pocket depth >7 mm, clinical attachment loss of >5 mm, grade III/IV furcation involvement, grade II/III mobility with the presence of Miller class III/IV gingival recession, are selected for sub-group B.

Clinical examination and parameters

All the patients were classified into three groups based on the American Academy of Periodontology 2018 classification. All the teeth were tested for pulp vitality status using the electronic pulp tester (EPT), and the following clinical parameters were recorded.

Probing Pocket Depth

University of North Carolina (UNC) - 15 probes were used to measure the probing pocket depth of the tooth at six sites - mesiobuccal, mid-buccal, distobuccal, distolingual, mid-lingual, and mesiolingual sites. The probing pocket depth was measured as the distance from the gingival margin to the base of the periodontal pocket. All the measurements were rounded off to the nearest millimeter.

Clinical Attachment Level

A UNC-15 probe was used to measure the clinical attachment level at six sites of the selected teeth: mesiobuccal, mid-buccal, distobuccal, distolingual, mid-lingual, and mesiolingual sites. The clinical attachment level was measured as the distance from the cementoenamel junction to the base of the periodontal pocket. All the measurements were rounded off to the nearest millimeter.

Gingival Recession

Gingival recession was measured with the UNC-15 probe as the distance from the cementoenamel junction to the gingival margin.

Furcation Involvement

Furcation involvement was recorded based on Glickman’s classification. Nabers probe was used to measure the horizontal component of the furcation. A UNC-15 probe was used to measure the vertical component of the furcation.

Radiographic evaluation

A radiovisuographic X-ray using the paralleling technique was obtained from all the patients included in the study. The remaining bone level with respect to the furcation area and the adjacent interproximal regions was recorded.

Extraction

All the extractions were performed by a single investigator. After recording all the required data, local anaesthesia (2% lignocaine with 1:80,000 adrenaline) was administered through infiltration with respect to the tooth intended for extraction. Atraumatic extraction of the tooth was done using elevators and forceps with minimal flap elevation. The socket was then debrided and packed with gauze. Post-operative instructions were given. Antibiotics (Cap Amoxicillin 500 mg BID, Tab Metronidazole 400 mg BID, Tab Paracetamol TID (after food), and Tab Pantoprazole 40 mg OD (half an hour before food)) were prescribed for three days. The patients were asked to report after two weeks for review.

Histopathological examination

After extraction, all the teeth were gently washed off from the blood and debris without any damage to the tooth specimen. The teeth were then transferred to a freshly prepared neutral buffered formalin (NBF) solution. It was then allowed to fix for about 24-30 hours. The teeth were then taken out and proceeded with decalcification. A freshly prepared formal nitric acid solution using 10 ml of formalin, 80 ml of distilled water, and 10 ml of nitric acid was used for decalcification. The solution was replaced with a fresh solution every day until decalcification was achieved. The decalcification endpoint was tested using physical and chemical methods such as the needle prick test and the bubble test. After decalcification, the teeth were sectioned longitudinally. In some multirooted teeth where the longitudinal sectioning of the teeth was not achieved, cross-sections were made.

The decalcified teeth were then processed using an alcohol solution (increasing grades of isopropyl alcohol), dehydrated using a xylene solution, and embedded in paraffin wax. Once the wax blocks were made, the tissue or teeth were sectioned using a microtome to a thickness of 3 to 4 µm. Before staining, the sections were deparaffinized using xylene and alcohol. Hematoxylin and eosin stains were used to stain all the tissue sections. The sections were then mounted and viewed under a microscope and analyzed under 10× to 40× magnification.

## Results

All the subjects underwent a thorough periodontal examination prior to extraction, and the clinical parameters were recorded for the control and study groups. The mean difference between the study groups was calculated, and it is depicted in Table 1.

**Table 1:** Mean difference of clinical parameters in the study groups. SD: standard deviation; group A: teeth extracted due to periodontitis without any gingival recession; group B: teeth extracted due to periodontitis with gingival recession.

Parameter	Group A (mean ± SD)	Group B (mean ± SD)
Probing pocket depth	8.42 ± 2.13	8.62 ± 1.58
Clinical attachment level	8.42 ± 2.13	15.02 ± 1.87
Recession	--	3.46 ± 0.51

The mean difference in PPD was found to be 8.42 ± 2.13 in group A and 8.62 ± 1.58 in group B subjects. The CAL was found to be 8.42 ± 2.13 and 15.02 ± 1.87 in group A and group B subjects, respectively. The mean difference of gingival recession was 3.46 ± 0.51 in group B subjects. 

**Figure 1:**
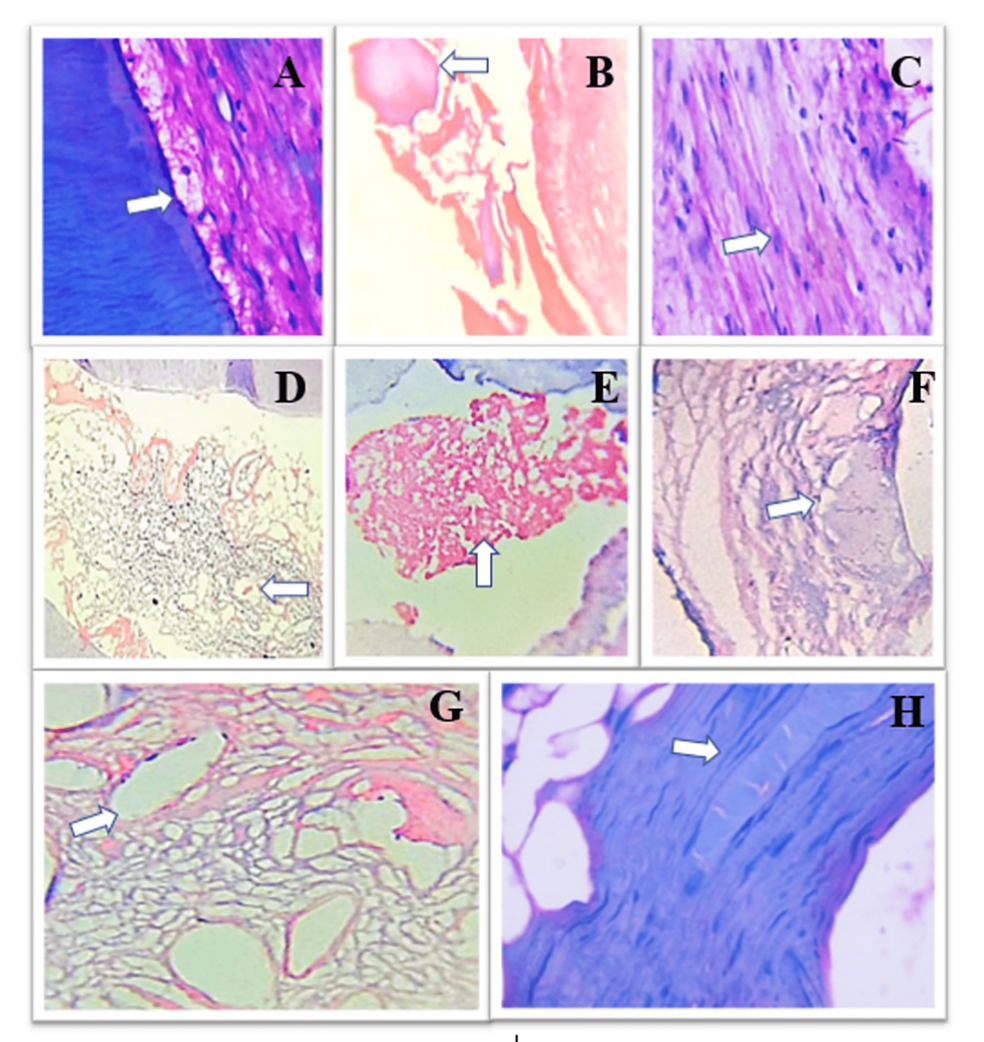
Histopathological pulpal changes in the study specimens. (A) Odontoblastic integrity (40×); (B) pulp stones (40×); (C) inflammation (40×); (D) edema (10×); (E) necrosis (10×); (F) atrophied blood vessels (10×); (G) dilated blood vessels; (H) fibrosis (40×).

After extraction and histopathological processing, the specimens were evaluated, and scoring was given for each criterion based on the presence of inflammation, fibrosis, edema, odontoblastic integrity, status of blood vessels, necrosis, and presence of pulp stones. Histopathological evaluation was done with various criteria for multirooted teeth in longitudinal and cross sections as shown in Figure 1.

**Figure 2:**
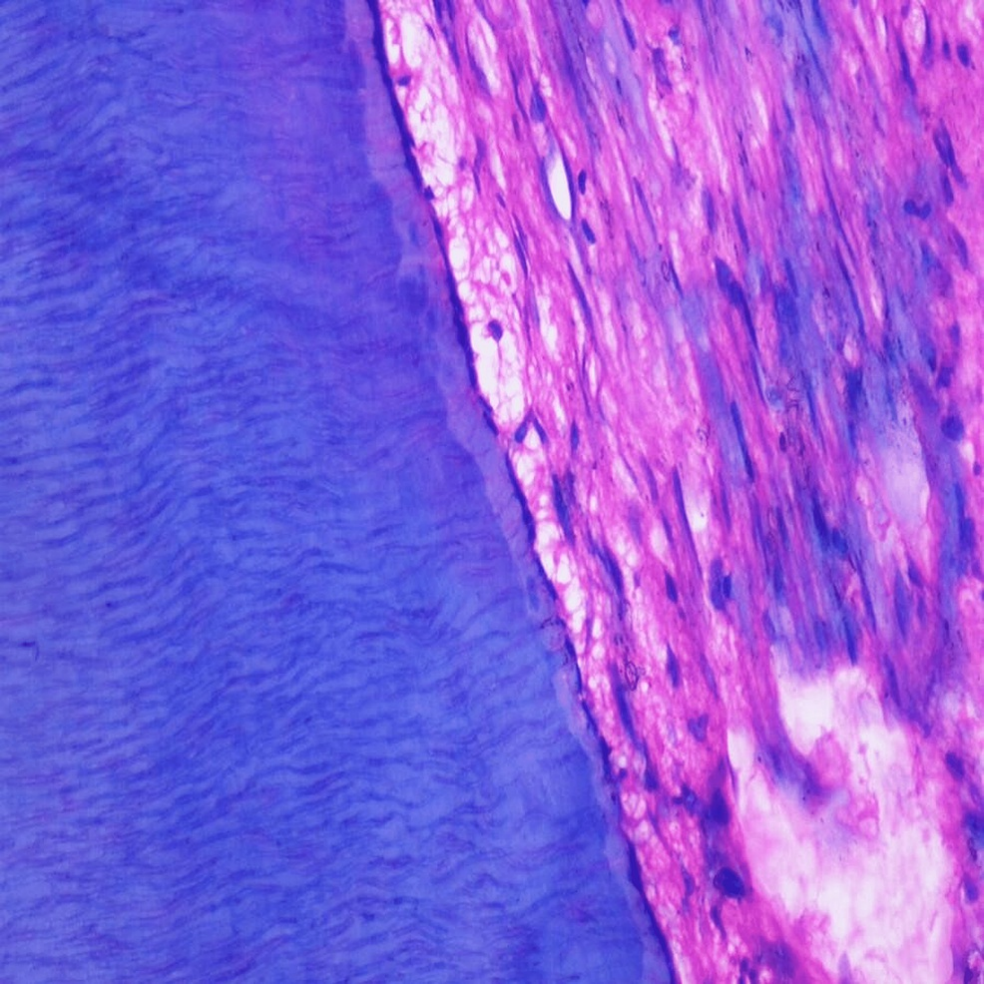
Histopathological picture of the control group specimen. Magnification - 40×.

**Figure 3:**
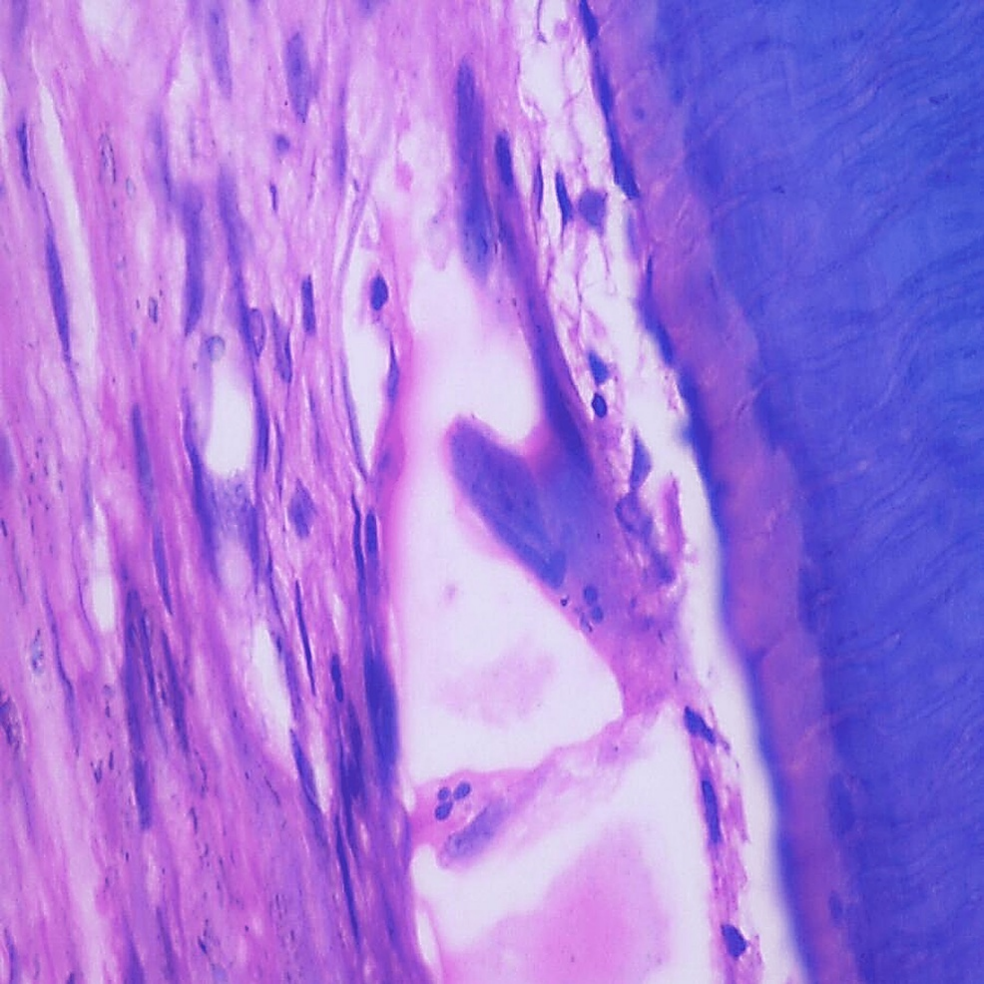
Histopathological picture of the control group specimen. Magnification - 40×.

Histopathological evaluation of the control group was done, and normal pulpal tissue with blood vessels, odontoblastic cells, fibroblasts, and inflammatory cells were observed majorly, as in Figures 2-3.

**Figure 4:**
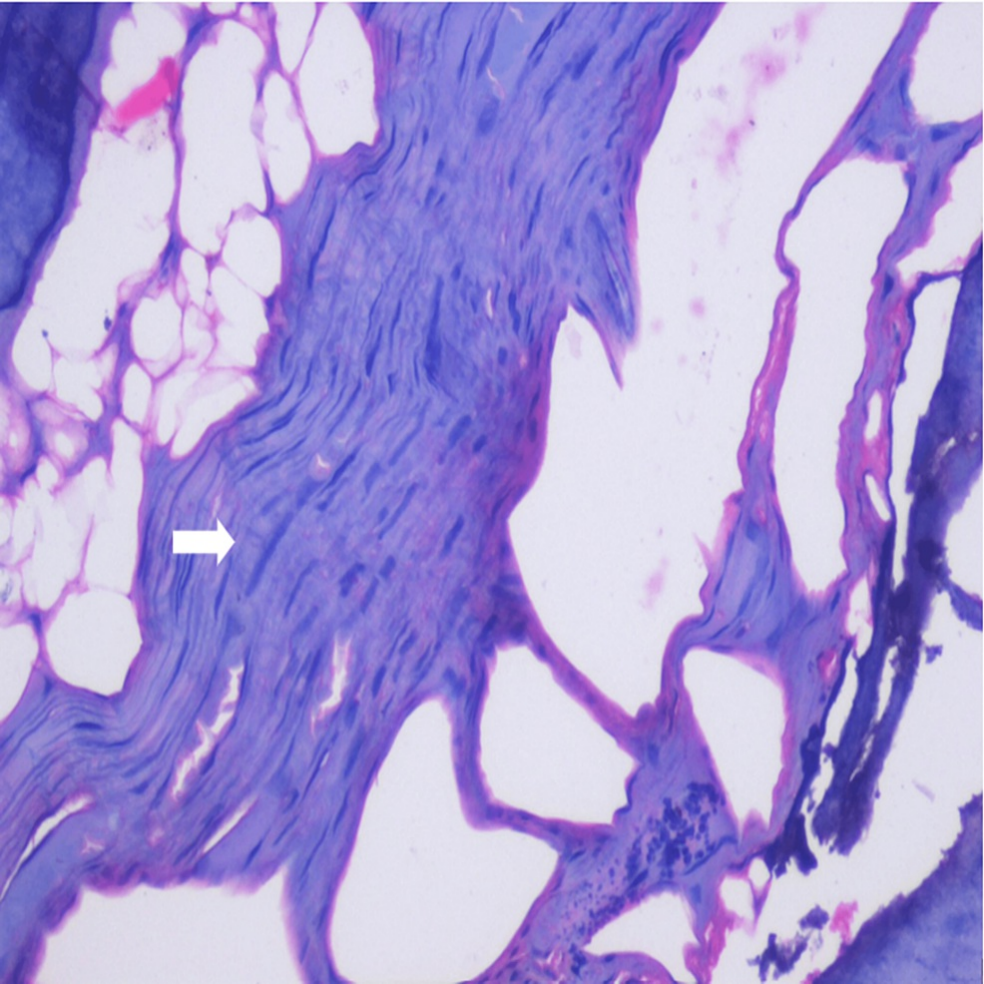
Histopathological picture of subgroup A depicting severe fibrosis in the pulp. Magnification - 40×.

Histopathological evaluation of subgroup A (advanced periodontitis without gingival recession) with the presence of severe fibrosis in the pulp is depicted in Figure 4.

**Figure 5:**
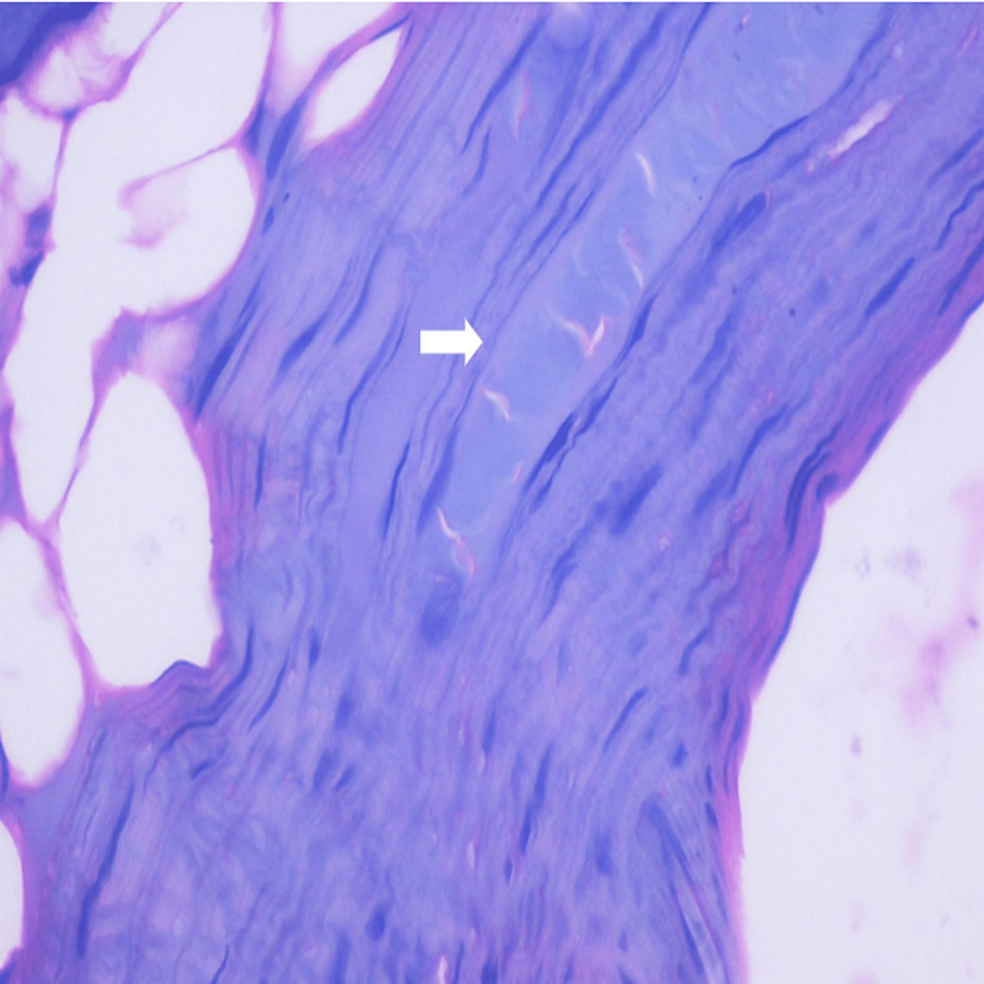
Histopathological picture of subgroup B with the presence of fibrosis turning into metaplastic calcification in the pulp. Magnification - 40×.

The histopathological evaluation of subgroup B (advanced periodontitis with gingival recession) with the presence of fibrosis turning into metaplastic calcification in the pulp is depicted in Figure 5.

These criteria were then analyzed using the chi-square test, and the results are shown in Table 2.

**Table 2:** Histopathological evaluation of the study and the control groups. N: number of teeth; %: percentage; p-value <0.05, is considered statistically significant.

Evaluation criteria	Parameter	Control (N)	%	Group A (N)	%	Group B (N)	%	p-value
Inflammation	Nil/absent	16	80	5	25	1	5	0.000*
	Mild	4	20	8	40	5	25	
	Moderate	0	0	4	20	7	35	
	Severe	0	0	3	15	7	35	
Fibrosis	Nil/absent	15	75	2	10	0	0	0.000*
	Mild	5	25	6	30	3	15	
	Moderate	0	0	5	25	7	35	
	Severe	0	0	7	35	10	50	
Edema	Nil/absent	16	80	0	0	0	0	0.000*
	Mild	4	20	10	50	4	20	
	Moderate	0	0	7	35	9	45	
	Severe	0	0	3	15	7	35	
Odontoblastic integrity	Present	20	100	12	60	3	15	0.000*
	Absent	0	0	8	40	17	85	
State of blood vessels	Normal	19	95	0	0	0	0	0.000*
	Dilated	1	5	17	85	12	60	
	Atrophic	0	0	0	0	0	0	
	Dilated/atrophic	0	0	3	15	8	40	
Necrosis	Present	0	0	14	70	18	90	0.000*
	Absent	20	100	6	30	2	10	
Pulp stones	Absent	20	100	3	15	0	0	0.000*
	Mild	0	0	8	40	6	30	
	Moderate	0	0	8	40	7	35	
	Severe	0	0	1	5	7	35	

All three grades of inflammation - mild, moderate, and severe - were observed in all the teeth of groups A and B. It was severe in 20% of group A specimens and 35% of group B specimens. Fibrosis was mild in the control group, whereas it was severe in 35% of group A and 50% of group B specimens. Edema was found in the control as well as in the study groups. It was found to be 50% mild, 35% moderate, and 15% severe in group A, and 45 % moderate and 35 % severe in group B specimens. Odontoblastic integrity was present in all the controls and 60% of group A specimens. Its integrity was not found in 40% of group A and 85% of group B subjects. The blood vessels of the pulp in group A and group B showed both dilatation and atrophy. It was dilated in 85% of group A specimens and 60% of group B specimens. The dilatation along with atrophy was seen in 15 % of group A and 40% of group B specimens. Necrosis of pulpal tissue was found in 70% of group A and 90% of group B specimens. Pulp stone was the common finding seen in the case group, and it was found to be moderate in 40% and 35% of group A and group B specimens, respectively. It was severe in 35% of group B. The P-value was found to be 0.000 for all the parameters and was statistically significant. Pulp stones and necrosis were absent in the control group, indicating no significant changes in the pulp.

The results showed that there were mild to moderate pulpal changes in the periodontitis group without a gingival recession (group A) and moderate to severe pulpal changes in the periodontitis group with a gingival recession (group B). Our study findings support the finding that there was a significant presence of histopathological changes in the pulp of periodontally affected/weakened teeth.

## Discussion

Previously done studies, correlating pulpal involvement with advanced periodontitis have only analyzed the effect of periodontitis and its severity in relation to pulp vitality. Gingival recession, tooth mobility, and furcation involvement, distinguishing maxillary and mandibular molars, to evaluate the changes associated with the trifurcation and the bifurcation involvement, as additional study parameters have not been researched previously. All these parameters were included in this study to bring forward a new perspective in the diagnosis, prognosis, and treatment planning of Perio-Endo lesions.

The current study aimed to assess the histopathological effects on pulp in teeth with periodontitis and its correlation with the presence of lateral and accessory canals. All 60 specimens in the study group, had lateral and accessory canals. We found that teeth affected by periodontitis have significant changes in pulp vitality due to the spread of infection to the pulp via the lateral and accessory canals, as in the case of multirooted teeth. This finding was relevant to a study done by Zuza et al. in 2012, where they evaluated the status of the pulp in 25 teeth affected by periodontitis, and histopathological analysis revealed three groups showing normal features, and the radicular pulps showed variable levels of reactive dentin, fibrosis, dystrophic mineralization, atrophy, and mononuclear inflammatory infiltrate [8]. They concluded that the gradual progression of chronic periodontitis could result in changes in the radicular pulp.

Similarly, Sheykhrezaee et al. in 2007 [9] and Caraivan et al. in 2012 [10] evaluated histopathological changes similar to the above study and concluded that pulpal changes are seen in periodontally affected teeth. They also added that advanced periodontitis could result in fibrosis and diffuse calcifications of dental pulp, which in turn might endanger root canal therapy. Our study also revealed a significant amount of fibrosis in teeth affected by periodontitis than those associated with gingival recession.

Rathod et al. noted that severe chronic periodontitis can affect dental pulp based on their study findings [11]. The cumulative effect of periodontal disease, root caries, and involved lateral canals damages the pulpal tissue and results in calcifications, resorption, and inflammation, but total disintegration is a certainty only when all main apical foramina are involved. This was in accordance with our study results, where severe pulpal calcifications and inflammation were seen when the teeth with gingival recession were compared to the group where gingival recession was not present.

Gautam et al. observed in their study that periodontally affected teeth resulted in pulpal calcifications and partial necrosis of the pulp [12]. They concluded that in the presence of moderate to severe chronic periodontitis, degenerative changes such as inflammation, fibrosis, edema, calcification, and necrosis were observed in variable degrees. In our study, the group with periodontitis and gingival recession showed an increased intensity of inflammation, edema, fibrosis, necrosis, and pulp stones when compared to the tooth where gingival recession was absent, indicating that severe periodontitis increased the occurrence of degenerative changes in the pulp.

Kulal et al. did a similar study as others and evaluated the histopathological changes that revealed periodontitis can induce changes in the dental pulp; considerations have to be given to the involved teeth for the depth of the pocket and severity of periodontal involvement for proper treatment planning and management [13].

Tan et al. evaluated the pulp status in the teeth of severely affected chronic periodontitis subjects. Sixty teeth were evaluated both clinically by electrical pulp testing and histopathologically for vitality [14]. They concluded that teeth affected by advanced periodontitis exhibited worse pulpal conditions with a decreased height of the residual periodontal membrane. The results of electric pulp testing were not completely representative of the histopathologic results in advanced periodontitis.

The odontoblastic integrity was evaluated in our study, and it showed that as the severity of periodontitis increased, the odontoblastic integrity was lost. This was relevant to a study done by Riccucci et al., where they stated that the pulp exhibited a detectable reactive response when the cementum coverage was lost or when the periodontal pocket extended to the root apex [15]. They also concluded that in some teeth, even before the periodontal disease reaches the apical root segment, the pulp displayed signs of severe inflammation and necrosis.

However, Sabeti et al. histopathologically evaluated teeth with advanced periodontitis and concluded that periodontal disease does not significantly affect pulp vitality. This was in contrast to our study results, where significant changes in the pulp were observed in moderate to severe periodontitis subjects [16].

## Conclusions

This study attempted to evaluate the effect of advanced periodontal disease and its symptoms, such as tooth mobility, furcation involvement, and gingival recession, on the vitality of pulpal tissues in multi-rooted teeth. The results obtained confirmed that periodontal disease extending to the furcation areas can indeed have an effect on the vitality of the tooth in the long term. Additionally, the presence of gingival recession can open the lateral and accessory canals, thereby serving as a pathway for the oral microorganism to gain access to the pulp and the root canal chambers.

Thus, we conclude that the presence of periodontal disease in multi-rooted teeth could lead to significant alterations in the pulp, thereby affecting its vitality. Hence, the treatment planning for periodontitis should include a vitality test of the dental pulp prior to the initiation of periodontal therapy in order to achieve long-term success for the patient.
